# Producing Photoactivated Room Temperature Phosphorescent Glass from Bamboo

**DOI:** 10.1002/advs.202512039

**Published:** 2025-09-25

**Authors:** Shaodi Zhang, Yingxiang Zhai, Jingyi Zhou, Jie Wu, Jian Gan, Boyan Jiang, Yuxiang Huang, Xiaoqi Zhao, Yahui Zhang, Zhijun Chen

**Affiliations:** ^1^ Research Institute of Wood Industry Chinese Academy of Forestry Beijing 100091 China; ^2^ State Key Laboratory of Utilization of Woody Oil Resource Northeast Forestry University Harbin China; ^3^ Key Laboratory of Bio‐based Material Science and Technology of Ministry of Education Northeast Forestry University Harbin China; ^4^ National centre for archaeology Beijing 100013 China

**Keywords:** 3D luminescent architectures, bamboo, mechanical strength, photoactivatable, room‐temperature phosphorescent

## Abstract

Developing sustainable, mechanically robust photoactivated room‐temperature phosphorescent (RTP) glasses is critical yet challenging. Such materials, B‐glass, are developed by infiltrating epoxy resin into a delignified bamboo framework, achieving exceptional mechanical strength (tensile: 133 MPa; impact: 55.6 kJ·m^−^^2^). Initial RTP emission in B‐glass is quenched by residual oxygen but can be photoactivated via UV irradiation (365 nm), where triplet excitons consume trapped oxygen, extending phosphorescence lifetime from 21.1 to 180.9 ms after 30 s exposure. Oxygen re‐diffusion under ambient air (7 h, dark) reversibly quenches emission, enabling dynamic on/off switching. Leveraging this behavior, B‐glass serves as a reprogrammable platform for 3D luminescent architectures and multilevel optical data storage. This work advances eco‐friendly RTP materials while offering a sustainable strategy for adaptive photonic technologies, balancing performance, environmental compatibility, and scalable fabrication.

## Graphical abstract

Room‐temperature phosphorescent (RTP) glass (B‐glass) is developed from natural bamboo. The as‐prepared B‐glass demonstrates unique photoactivated RTP characteristics and superior mechanical properties, which can serve as a reprogrammable platform for 3D luminescent architectures and multilevel optical data storage.

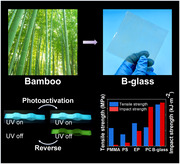

## 1 Introduction

Organic room‐temperature phosphorescent (RTP) materials have emerged as promising candidates for diverse applications such as optical displays,^[^ ^1^ ^]^ anti‐counterfeiting systems,^[^ ^2^ ^]^ bioimaging,^[^ ^3^ ^]^ scintillators,^[^ ^4^ ^]^ and radiative cooling technologies.^[^ ^5^ ^]^ To achieve efficient RTP emission from organic systems, two critical design principles must be satisfied: 1) Enhancement of intersystem crossing (ISC) from singlet to triplet excitons through optimized spin‐orbit coupling in chromophores, and 2) Facilitation of radiative transition from triplet excitons to the ground state via molecular rigidification strategies.^[^ ^6^, ^7^, ^8^ ^]^ These guiding principles have driven the development of various organic RTP materials in forms including molecular crystals,^[^ ^9^ ^]^ supramolecular assemblies,^[^ ^10^ ^]^ metal‐/covalent‐/hydrogen‐bonded organic frameworks (MOFs/COFs/HOFs),^[^ ^11^, ^12^, ^13^ ^]^ and polymer composites,^[^ ^14^ ^]^ fabricated as powders, films, foams, and structural components.^[^ ^8^, ^15^, ^16^ ^]^ Notably, photoactivated organic RTP glasses have attracted particular interest as next‐generation optical platforms for photonic and electronic devices due to their unique stimulus‐responsive properties.^[^ ^17^, ^18^, ^19^ ^]^

However, current organic RTP glasses predominantly rely on petroleum‐derived precursors, raising concerns regarding environmental sustainability and toxicity during synthesis.^[^ ^20^, ^21^ ^]^ Furthermore, these materials typically exhibit inherent mechanical limitations such as brittleness and low deformation resistance, significantly constraining their practical utility in demanding applications.^[^ ^22^ ^]^

**Figure 1:**
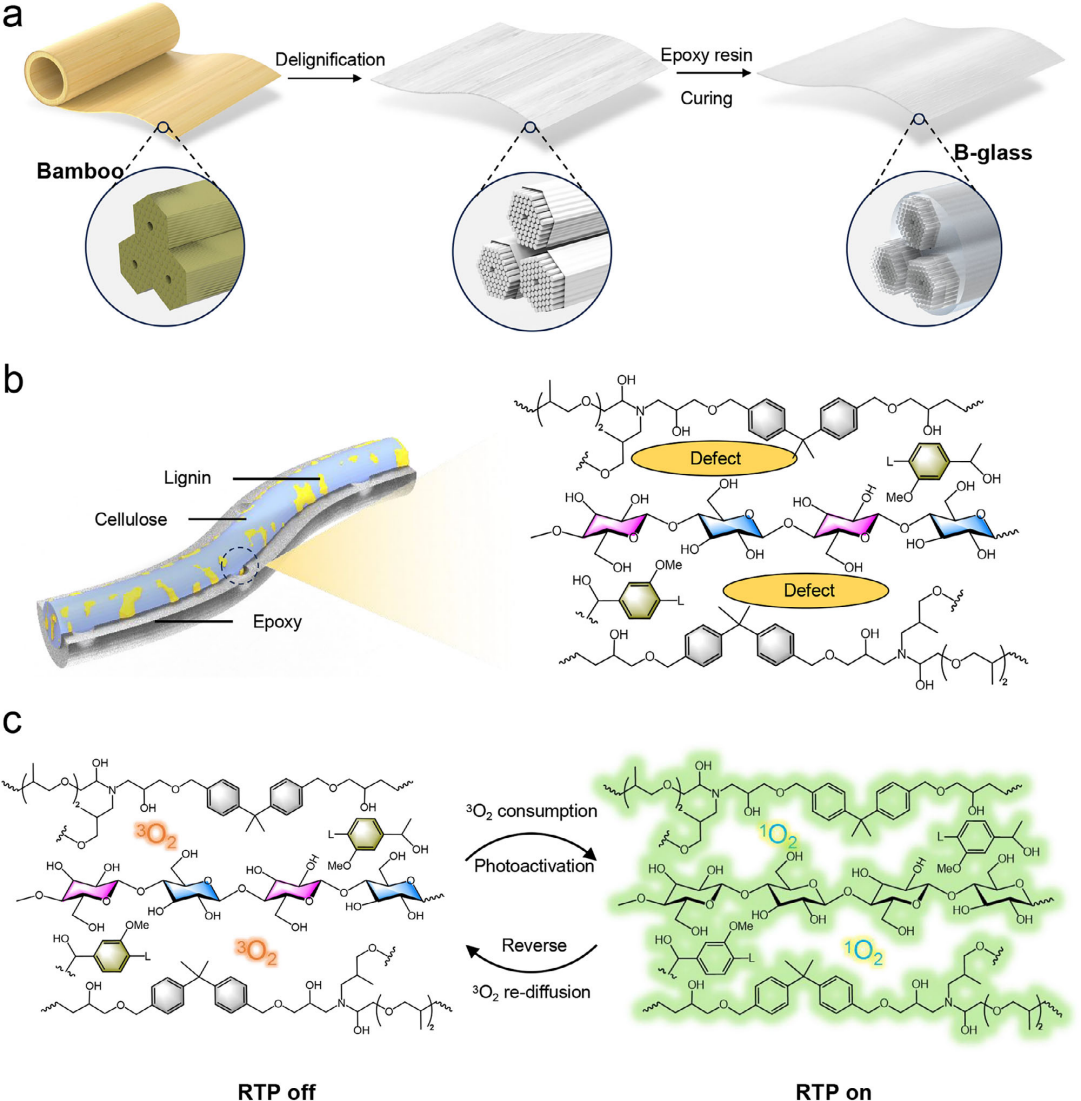
Schematic illustration of the preparation and structure of B‐glass. a) Preparation of B‐glass; b) Impregnated epoxy resin exhibited weak intermolecular interaction with delignified bamboo, causing structural defects. c) Photoactivated RTP properties of B‐glass.

In response to these challenges, recent research has focused on exploiting renewable biomass resources for RTP material development.^[^ ^2^, ^6^, ^8^ ^]^ While materials derived from wood,^[^ ^23^, ^24^ ^]^ bamboo,^[^ ^25^ ^]^ hemicellulose,^[^ ^26^, ^27^ ^]^ cellulose,^[^ ^28^, ^29^, ^30^ ^]^ lignin,^[^ ^31^, ^32^, ^33^ ^]^ and natural phenolics^[^ ^34^, ^35^ ^]^ have demonstrated RTP capabilities, the creation of sustainable photoactivated RTP glasses from biomass remains an underexplored frontier. In this work, we present a mechanically robust photoactivated RTP glass (termed B‐glass) synthesized from bamboo biomass. The fabrication process involved controlled delignification to partially remove lignin components, followed by epoxy resin (EP) infiltration of the modified bamboo matrix (**Figure** **1**a). The B‐glass exhibited exceptional mechanical properties with a tensile strength of 133 MPa and impact resistance of 55.6 kJ·m^−^^2^. Structural analysis revealed that weak interfacial interactions between the epoxy matrix and delignified bamboo induced microstructural defects (Figure 1b), enabling environmental oxygen interaction with triplet excitons and resulting in suppressed initial RTP emission. Remarkably, the B‐glass demonstrated photoactivatable RTP characteristics, achieving an extended phosphorescence lifetime of 180.9 ms under UV excitation (Figure 1c).

## 2 Results and Discussion

### 2.1 Preparation and Characterization of B‐Glass

**Figure 2:**
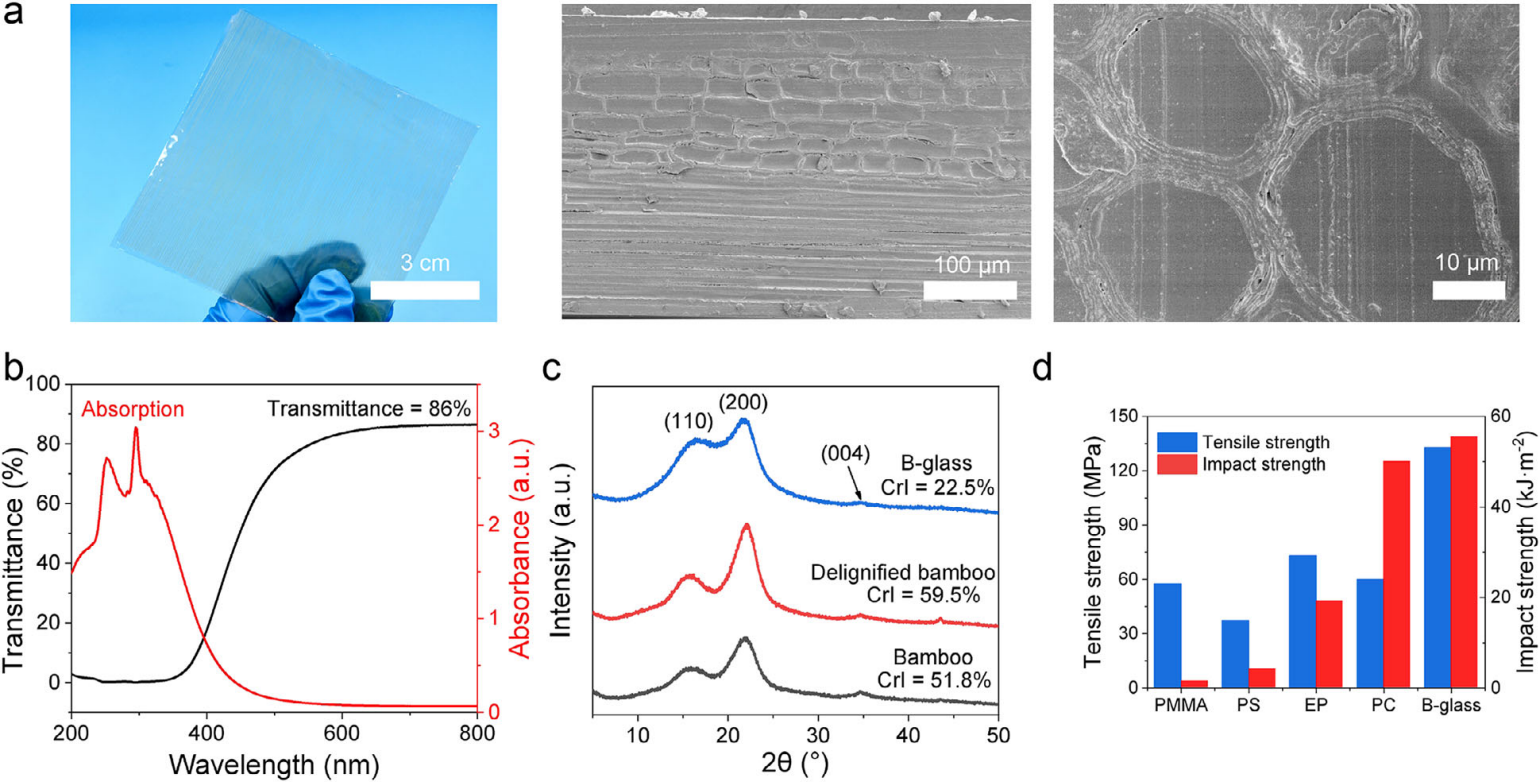
Morphological and structural characterizations, and mechanical properties of samples. a) Optical appearance image, longitudinal and cross‐section microscopic images of B‐glass; b) Adsorption spectra and transmittance of B‐glass; c) Powder X‐Ray diffraction patterns of samples; d) Comparison of the tensile and impact strength of B‐glass with other commercial organic glasses.

Compositional analysis of delignified bamboo was conducted, revealing constituent percentages of 85.87% cellulose, 13.95% hemicellulose, and 0.18% lignin. In contrast, untreated bamboo contained 48.39% cellulose, 25.16% hemicellulose, and 26.45% lignin, confirming substantial lignin removal during processing. The delignification process not only modified chemical composition but also induced significant morphological changes. Native bamboo comprises two distinct cell types: densely packed, thick‐walled fiber cells forming high‐strength macrofiber bundles, and thin‐walled parenchyma cells with hollow structures serving as a matrix (Figure S1, Supporting Information). Following acidic NaClO_2_ treatment, the bamboo veneer whitened while maintaining structural integrity, with macrofiber bundles developing cracks/voids and parenchyma cell separation (Figure S1, Supporting Information)‐a structural modification conducive to polymer impregnation. The delignified bamboo was subsequently infused with epoxy resin (EP) and cured to form B‐glass. Microstructural analysis demonstrated that cured EP filled intercellular gaps, lumens, and cell wall pores, creating a 3D interpenetrating network (**Figure** **2**a). This dense architecture contributed to B‐glass's exceptional optical transmittance and haze, with UV–vis spectra showing primary absorption in the ultraviolet range due to residual lignin (Figures 2b; S2, Supporting Information). X‐ray diffraction patterns retained characteristic cellulose peaks at 16.0° (110) and 21.8° (200) from native bamboo, though crystallinity decreased significantly (Figure 2c). This reduced crystallinity enhanced light transmission capabilities, further improving optical transparency. B‐glass demonstrated remarkable mechanical properties (Figure S3, Supporting Information), achieving a tensile strength of 133 MPa – surpassing conventional organic glasses including Poly(methyl methacrylate) (PMMA, 57.7 MPa),^[^ ^36^ ^]^ Polystyrene (PS, 37.5 MPa),^[^ ^37^ ^]^ EP (73.2 MPa),^[^ ^38^ ^]^ and Polycarbonate (PC, 60.0 MPa) (Figure 2d).^[^ ^39^ ^]^ Due to the well‐aligned fibers of bamboo, B‐glass exhibited anisotropic compressive strength (Figure S4, Supporting Information). Additionally, B‐glass can remain stable in environments ranging from room temperature to 200 °C, suggesting good thermal stability (Figure S5, Supporting Information).

### 2.2 Photoactivated RTP of B‐glass

**Figure 3:**
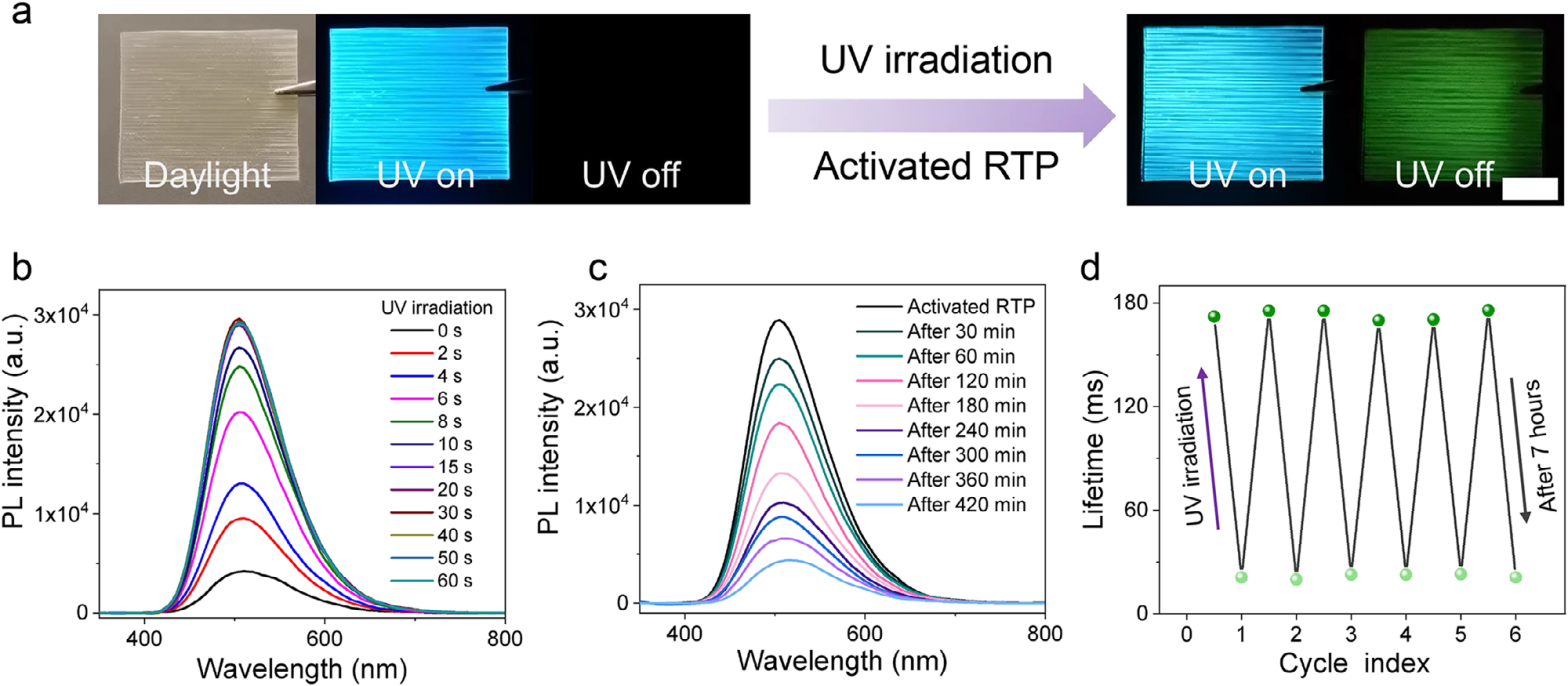
Optical properties of B‐glass. a) Images of B‐glass upon UV irradiation and after removing the UV excitation, scale bar = 1 cm; b) Phosphorescence spectra of B‐glass upon UV irradiation for different times; c) Phosphorescence spectra of B‐glass upon UV irradiation (black line) and phosphorescence spectra of B‐glass activated by UV irradiation in an ambient environment for different times; d) RTP lifetime of B‐glass upon UV irradiation and after switching off the UV light source.

The photoluminescent properties of B‐glass were systematically examined. Initial characterization revealed negligible room‐temperature phosphorescence (RTP) emission under ambient conditions (**Figures** **3**a; Video S1, Supporting Information). However, both RTP intensity and lifetime exhibited progressive enhancement during continuous UV excitation (365 nm, 170 mW·cm^−^^2^), demonstrating light‐activated behavior (Figures 3b; S6, Supporting Information). Furthermore, by adjusting the UV light intensity, B‐glass with varying photoactivation intensity and phosphorescence lifetime can be obtained (Figure S7, Supporting Information). Upon excitation, B‐glass shows fluorescence at 410 nm (Figure S8, Supporting Information) and RTP emission at 510 nm with a quantum yield of 4.1% (Figure S9, Supporting Information). Intriguingly, these photophysical parameters displayed reversible attenuation upon cessation of illumination (Figures 3c; S10, Supporting Information), maintaining cycle stability through multiple ON‐OFF transitions without significant lifetime degradation (Figure 3d). Since oxygen suppresses the phosphorescence of B‐glass, fully exposing the photoactivated B‐glass to an oxygen‐rich environment will quickly quench its phosphorescence, allowing it to completely return to its initial non‐luminescent state within 30 min (Figure S11, Supporting Information). This robust reversibility highlights the material's stable stimulus‐responsive characteristics. In addition, B‐glass exhibited good durability under a harsh environment. After an accelerated weathering test for 168 h, the color of B‐glass turned to brownish yellow, and B‐glass can still demonstrate photoactivated phosphorescence. And there is no significant reduction in phosphorescence intensity or duration after photoactivation (Figure S12, Supporting Information). Notably, the photoactivated lifetime of B‐glass maintains an almost unchanged lifetime after 3‐month storage under ambient conditions (Figure S13, Supporting Information). Besides epoxy resin, the composite material obtained by infiltrating PMMA into a delignified bamboo scaffold also exhibited photoactivated RTP. After 30 s of UV irradiation, its RTP lifetime increased from 34.9 to 66.5 ms (Figure S14, Supporting Information). Furthermore, we also compared the difference in phosphorescence lifetimes between B‐glass and the currently reported RTP materials (such as MOFs, carbon dots), B‐glass exhibits a comparable lifetime with many of these materials (Figure S15, Supporting Information).

### 2.3 Mechanism

To elucidate the origin of this photoactivated RTP, comparative studies were conducted with control samples. E‐glass, fabricated solely from cured epoxy resin without bamboo reinforcement, showed a complete absence of RTP activity under identical conditions (Figure S16, Supporting Information), confirming the essential role of delignified bamboo in the photophysical phenomenon. Subsequent analysis revealed that isolated delignified bamboo components exhibited intrinsic RTP emission upon UV excitation (Figure S17, Supporting Information), attributed to synergistic effects of residual lignin confinement and polysaccharide clustering‐induced emission.^[^ ^25^ ^]^ Notably, this inherent RTP displayed saturation behavior under prolonged UV exposure (Figure S17, Supporting Information), contrasting sharply with the progressive enhancement observed in B‐glass. These comparative results establish that interfacial interactions between the epoxy matrix and delignified bamboo constituents critically enable the unique photoactivation mechanism.

**Figure 4:**
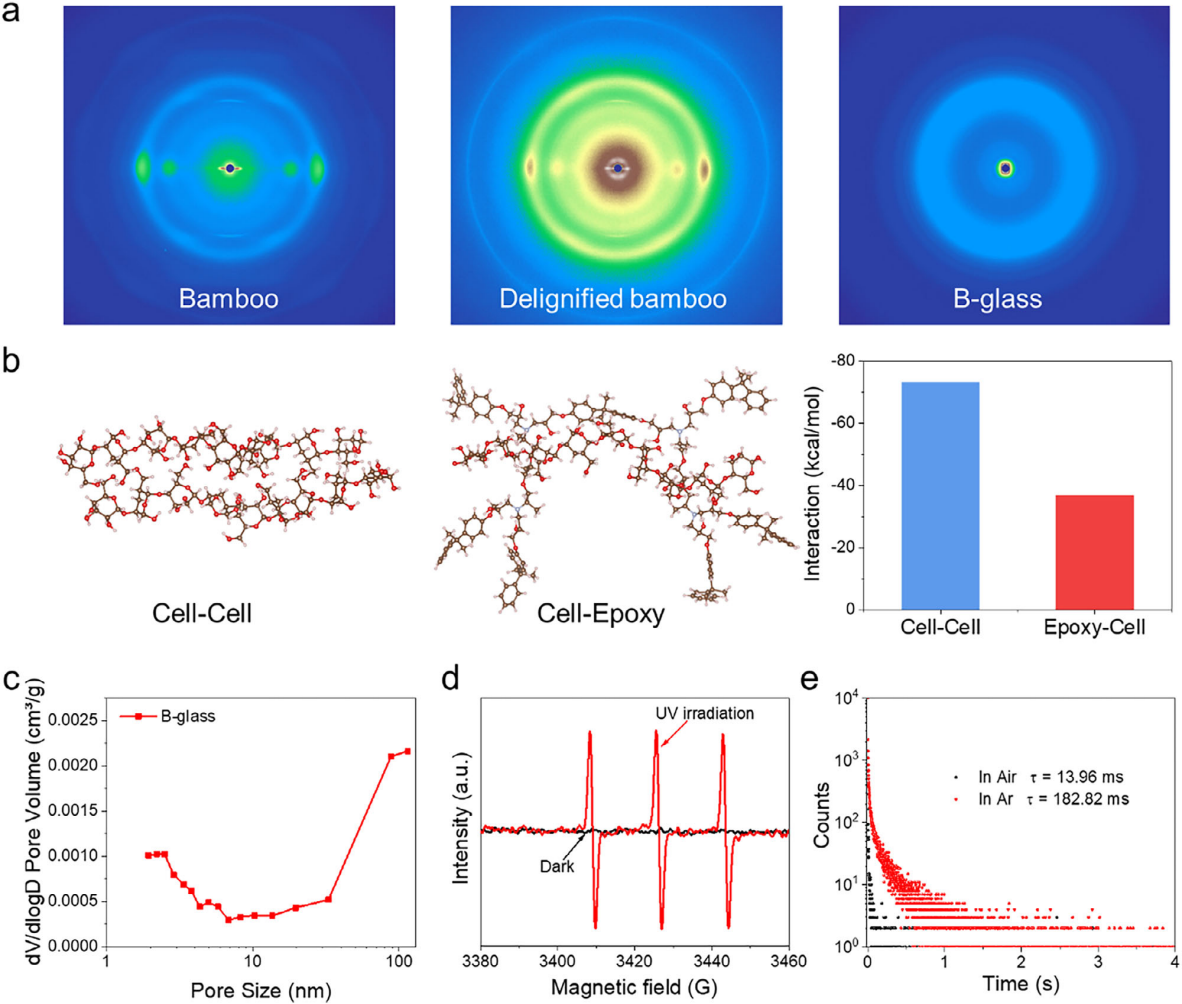
Mechanisms for the photoactivated RTP performances of B‐glass. a) 2D‐WAXS patterns of bamboo, delignified bamboo, and B‐glass, b) Theoretical simulation of the interaction between ‐celluloses (cell‐cell) and Epoxy‐cellulose (Epoxy‐cell), and their binding energy; c) BJH mesopore size distribution derived from the adsorption branch of Nitrogen adsorption‐desorption isotherms; d) EPR spectra of B‐glass in the presence of TEMP in the dark and upon UV irradiation at air atmosphere; e) Initial RTP lifetime of B‐glass in Ar and air.

To elucidate interfacial interactions, we performed 2D wide‐angle X‐ray scattering (2D WAXS) analysis. Both native and delignified bamboo exhibited four distinct Bragg reflections along the equatorial axis, while B‐glass showed no crystalline signatures attributable to its epoxy‐dominated amorphous matrix (**Figure** **4**a). Gaussian deconvolution of scattering profiles (10 °–30 °) resolved four components corresponding to (1–10), (110), and (200) lattice planes, along with an amorphous phase (Figure S18 and Table S1, Supporting Information). Notably, B‐glass demonstrated reduced crystallite dimensions along the (200) direction and expanded interlayer spacing relative to native/delignified counterparts, suggesting lattice distortion induced by epoxy infiltration. Density functional theory (DFT) calculations revealed weaker interfacial interactions between epoxy chains and cellulose (*ΔE* = −36.91 eV) compared to native cellulose–cellulose complexes (*ΔE* = −73.51 eV) (Figure 4b). These findings demonstrate that epoxy resin successfully penetrates the crystalline domains of delignified bamboo, modifying crystal architecture and establishment of weaker interfacial interaction in B‐glass composite.

This weakened interfacial bonding induced structural defects, as further evidenced by N_2_ gas adsorption, detecting abundant 2–10 nm mesopores in B‐glass (Figure 4c). These nanopores facilitated ambient oxygen/humidity permeation, effectively quenching triplet excitons and suppressing initial RTP. To further verify the effect of pores on the phosphorescence of B‐glass, samples with varying porosity were prepared. It was found that the increased defects and BET surface area led to higher oxygen permeability in the samples, consequently requiring a longer photoactivation time (Table S2, Supporting Information). In addition, as the thickness of B‐glass increases, the time required for photoactivation becomes longer, indicating the micropores within epoxy resin also contribute to the quench of triplet excitons. The RTP lifetime upon photoactivation of B‐glass remains unaffected when changing thickness (Figure S19, Supporting Information). Intriguingly, B‐glass maintained detectable RTP emission even under water (Figures S20 and S21, Supporting Information), confirming moisture resistance while implicating oxygen as the dominant quenching agent. The photoactivation mechanism was investigated through controlled atmosphere experiments. A proposed model suggests UV‐induced oxygen consumption via triplet exciton‐mediated sensitization to singlet oxygen (^1^O_2_). Electron paramagnetic resonance (EPR) spectroscopy using 2,2,6,6‐tetramethylpiperidine (TEMP) as a spin trap confirmed this hypothesis: strong TEMPO signals emerged under UV/air conditions (*g* = 2.006) but remained absent in dark or argon environments (Figures 4d; S22, Supporting Information). Parallel RTP measurements revealed a 13‐fold lifetime enhancement in argon (182.8 ms vs 14.0 ms in air; Figures 4e; S23, Supporting Information), conclusively identifying molecular oxygen as the primary quencher. Material permeability studies provided further validation. B‐glass exhibited high oxygen transmission rates (18.37 cm^3^·mm^−^^2^·24 h^−1^·0.1 MPa^−1^), contrasting sharply with polyvinyl alcohol PVA‐bamboo composites (4.72 cm^3^ cm^−^^3^·mm^−^^2^·24 h^−1^·0.1 MPa^−1^) that demonstrated immediate RTP emission without activation (Figures S24 and S25, Supporting Information). This disparity underscores the critical role of PVA's hydroxyl groups in forming oxygen barrier networks. The photophysical behavior of B‐glass follows an oxygen‐regulated RTP activation mechanism: The suppressed initial RTP emission originates from triplet exciton quenching by molecular oxygen permeating through the material's suboptimal oxygen barrier network. Following UV irradiation, triplet excitons participate in an energy transfer cascade that both consumes residual O_2_ and generates metastable singlet oxygen (^1^O_2_). This dual process progressively depletes the quencher population while establishing a transient oxygen‐depleted microenvironment, thereby activating persistent phosphorescence. The system demonstrates dynamic reversibility, as ambient oxygen rapidly rediffuses into the matrix upon illumination cessation, reinstating the quenched ground state through non‐radiative energy dissipation pathways.

### 2.4 Applications

**Figure 5:**
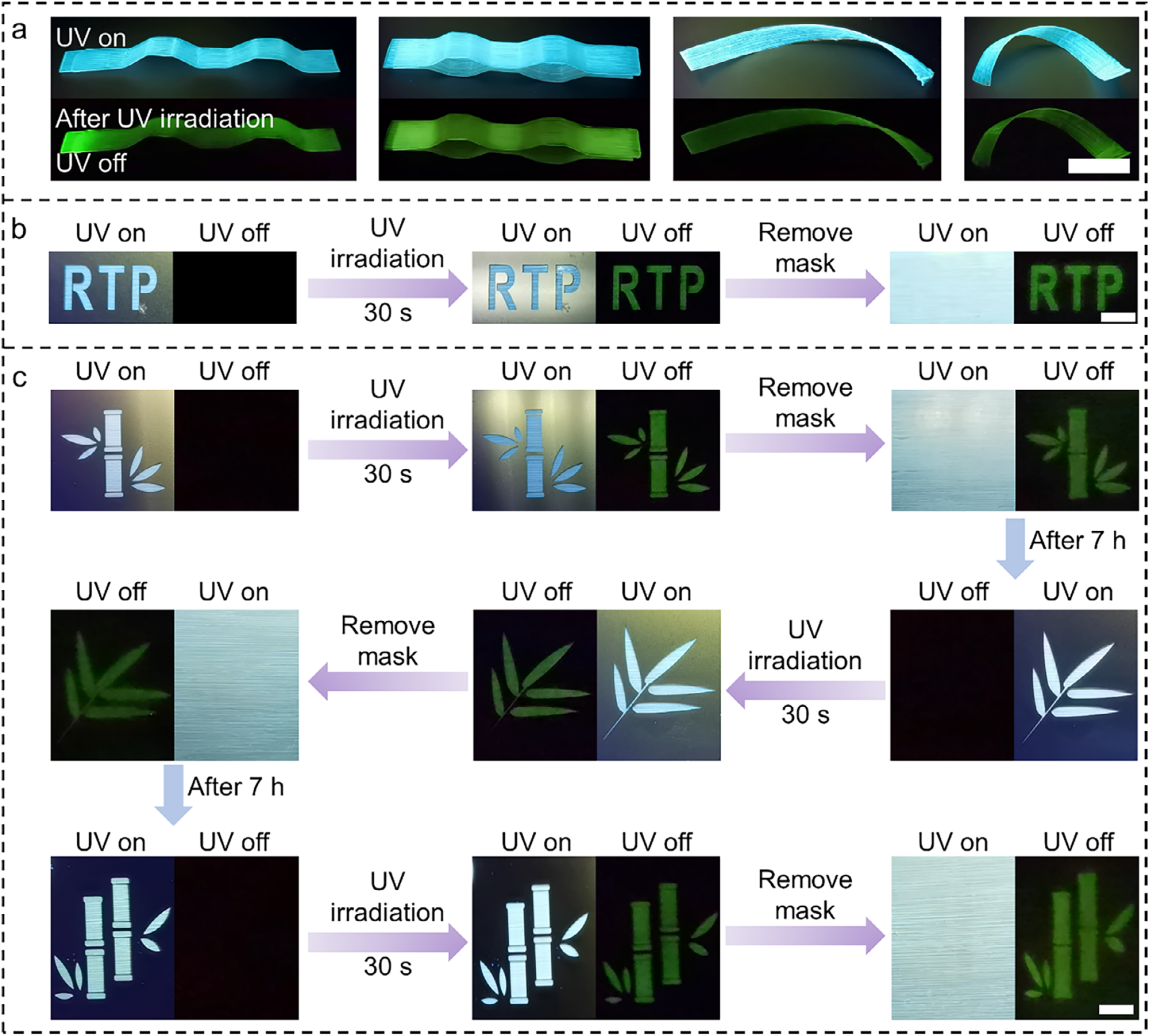
Applications. a) Photograph of different shapes of B‐glass and its phosphorescence after UV activation; b) Fabricating RTP images with B‐glass using UV activation, scale bar = 1 cm; c) Recycling (one bamboo‐leaf‐two bamboo) RTP images in B‐glass using UV activation.

Capitalizing on its exceptional processability, B‐glass demonstrates versatile fabrication potential, enabling the creation of 3D architectures through molding techniques (Figures S26 and S27, Supporting Information). Crucially, these engineered structures retain their photoactivated RTP characteristics (**Figure** **5**a).

The stimulus‐responsive nature of B‐glass enables novel applications in dynamic information encryption. Implementing a custom‐designed photomask during UV irradiation (*λ* = 365 nm) creates localized activation of RTP patterns. Initial inspection under ambient or UV‐illuminated conditions reveals no discernible features (Figure 5b). However, upon illumination cessation, high‐contrast latent patterns emerge through differential RTP persistence in irradiated zones. This optical memory effect demonstrates environmental responsiveness through gradual pattern fading, achieving complete erasure within 7 h through ambient oxygen diffusion (Figure 5c). The system supports cyclic rewriting through sequential mask‐aligned irradiation (Figure 5c). This fatigue‐resistant reconfigureurability stems from the material's reversible oxygen exchange mechanism without permanent photochemical degradation.

## 3 Conclusion

In summary, we successfully fabricated room‐temperature phosphorescent (RTP) glass (B‐glass) derived from natural bamboo. The as‐prepared B‐glass demonstrated superior mechanical properties, achieving a tensile strength of 133 MPa and impact resistance of 55.6 kJ·m^−^^2^. Notably, the material exhibited unique photoactivated RTP characteristics. Initial characterization revealed no intrinsic RTP emission, which we attribute to oxygen diffusion quenching through structural defects in the glass matrix. However, upon UV irradiation for 30 s, the B‐glass displayed distinct RTP emission with a lifetime of 180.9 ms. This phenomenon originates from UV‐induced triplet excitons that effectively sensitize incorporated oxygen molecules to form singlet oxygen (^1^O_2_). Intriguingly, the RTP emission underwent reversible quenching through oxygen rediffusion after ceasing UV exposure for 7 h, yet could be fully regenerated through subsequent UV excitation for 30 s. This dynamic optical behavior enables the development of B‐glass‐based rewritable optical chips for information storage applications. Furthermore, from an environmental friendliness, deep eutectic solvents (DES) could be employed in future experimental designs to replace NaClO_2_/H_2_O_2_ for lignin removal. Considering its facile fabrication process, cost‐effectiveness, and sustainable bamboo‐derived composition, we propose B‐glass as a highly promising candidate for next‐generation eco‐friendly photonic materials.

## 4 Experimental Section

### Preparation of B‐Glass

Natural bamboo veneer was dried in an 80 °C oven for 6 h until it reached a constant weight. Oven dried bamboo veneers were immersed in NaClO_2_ solution (2% w·w^−1^, pH = 4.4–4.5) and heated in a 55 °C water bath for 24 h. The solution was changed every 6 h. After that, the bamboo veneer samples were further treated by H_2_O_2_ solution (8% w·w^−1^) and heated in a 55 °C water bath for another 2 h until the samples turned white. Then the delignified bamboo were washed with deionized water until neutral and then stored in ethyl alcohol (> 99.7%). The delignified bamboo was immersed in epoxy resin (resin: hardener = 10: 3) under vacuum (100 Pa) for 10 min. This process was repeated for three times. The epoxy impregnated delignified bamboo was molded and cured for 48 h to obtain B‐glass.

### Preparation of E‐Glass

Epoxy resin (resin: hardener = 10: 3) was stored in a Polytetrafluoroethylene film‐coated glass container, cured for 48 h to obtain E‐glass.

### Preparation of PVA‐Bamboo Composites

Delignified bamboo was stored in water for 1 d then immersed in a 10 wt% PVA solution under vacuum (100 Pa) for 2 h. The PVA solution impregnation process was repeated for three times. The PVA impregnated delignified bamboo was stored in a glass container and dried in an ambient environment for 1 week to obtain PVA‐bamboo composites.

## Conflict of Interest

The authors declare no conflict of interest.

## Supporting information



Supporting Information

Supplemental Video 1

## Data Availability

The data that support the findings of this study are available in the supplementary material of this article.
