# Supplementary material for: Producing Photoactivated Room Temperature Phosphorescent Glass from Bamboo
Source: Adv Sci (Weinh). 2025 Sep 25;12(46):e12039. doi: 10.1002/advs.202512039 (PMC12697790; doi:10.1002/advs.202512039)
Supplement: Supplementary file 1 — Supporting Information [file ADVS-12-e12039-s002.docx]

Supporting Information

**Producing photoactivated room temperature phosphorescent glass from bamboo**

*Shaodi Zhang^a,#^, Yingxiang Zhai^b,c,#^, Jingyi Zhou^b,c^, Jie Wu^a^, Jian Gan^a^, Boyan Jiang^a^, Yuxiang Huang^a^, Xiaoqi Zhao^d^,* *Yahui Zhang^a,*^, Zhijun Chen^b,c*^*

^a^ Research Institute of Wood Industry, Chinese Academy of Forestry, Beijing, 100091, China.

^b^ State Key Laboratory of Utilization of Woody Oil Resource, Northeast Forestry University, Harbin, China.

^c^ Key Laboratory of Bio-based Material Science and Technology of Ministry of Education, Northeast Forestry University, Harbin, China.

^d^ National centre for archaeology, Beijing, 100013, China.

^#^ These authors contributed equally to this work.

^*^ Corresponding authors Emails: zhangyh0206@163.com (Y. Zhang); chenzhijun@nefu.edu.cn (Z. Chen);

Methods

Materials

Rotary-cut bamboo veneer (4-year Moso bamboo (*Phyllostachys edulis* (Carrière) J. Houzeau). Among the numerous varieties of bamboo, moso bamboo was reported to have the fastest growth rate in the world (maximum growth rate of 114.5 cm/day). Compared with other species of bamboo, moso bamboo is more widespread and cheaper, so we choose moso bamboo as the main research object in this article.) with a thickness of 300-400 μm was obtained from a local bamboo product industry in Zhejiang Province, China. Bisphenol-A epoxy resin (epoxy equivalent = 166-185 g·eq^-1^, epoxy value = 0.54-0.60) and a polyetheramine hardener (Amine value = 500 mg KOH·g^-1^) were provided by Jiulimei Electronic Materials Co., Ltd. Other chemicals including NaClO_2_ (AR), H_2_O_2_ (35 %), and ethyl alcohol (AR, > 99.7%) were purchased from Macklin Biochemical Co. Ltd., Shanghai, China. Polyvinyl alcohol (PVA105, 95%) was purchased from Kuraray Co., Ltd., Japan. 2,2,6,6-tetramethylpiperidine (TEMP, AR) was obtained from SigmaAldrich, USA. Deionized water used in this work was homemade.

Characterizations

The micro- morphologies of samples were recorded by a Field-emission scanning electron microscopy (FE-SEM, Hitachi S-4800, Hitachi, Japan). Fourier transform infrared (FTIR) spectra were measured on a FTIR spectrometer (Nicolet IS 10, Nicolet, USA) in the range of 4000-400 cm^-1^. The surface area and porous structure was determined by the physical adsorption of N_2_ at -196 °C using an Autosorb-iQ analyzer (BET; ASAP 2360, USA). The pore size distribution (PSD) of samples was calculated by Barrett-Joyner-Halenda (BJH) method. Powder X-ray Diffraction (XRD) patterns of samples were collected by using a X-ray Powder diffractometer (Bruker D8-Advanced, Germany) with Cu Ka radiation at 40 kV and 40 mA, the data were recorded in the 2θ range of 5-50 ° with an angular step size of 0.02 ° and a counting time of 8 s per step. Tensile strength was tested by an electronic universal mechanical testing machine (ETM, WANCE, China) using a 10 kN load cell with a crosshead speed of 2 mm·min^-1^ according to ASTM D638-96 standard. ‌Notched Charpy impact strength (1A type notch) was tested using an impact tester with a pendulum of 4 J energy according to GB/T1043.1-2008 standard. The cellulose, lignin and hemicelluloses compositions of the samples were measured using standard methods (Technical Association of Pulp and Paper Industry Standard Method T 222-om-83). The generation of singlet oxygen in B-glass under air and argon atmosphere was determined using electron paramagnetic resonance (EPR) analysis using a Bruker EMXplus-6/1 spectrometer (Bruker, Germany) with a 500 W Xenon-lamp as light source. Transmittance (T), haze and reflectance (R) were measured via the UV–vis Spectrometer Lambda 35 (PerkinElmer, USA) equipped with an integrating sphere. The oxygen permeability of B-glass and a PVA film was measured by differential-pressure method using a GTR-701M Gas Transmission Rate Tester (SYSTESTER, China) according to GB/T 1038-2000 standard. The diffusion cell area was 38.48 cm^2^.

Preparation of B-glass with various pore structure

For regulating the pore structure of B-glass, delignified bamboo was immersed in epoxy resin under vacuum treatment for various time (i.e., 5/10/30 min in total). Short vacuum time can not remove ethanol completely, therefore leaving defects in the bamboo structure and the interface between bamboo and epoxy resin. The epoxy impregnated delignified bamboo was molded and cured for 48 h to obtain B-glass 5min, B-glass 10min, and B-glass 30min (same as B-glass discussed in the manuscript).

Accelerated weathering test

The weathering test was conducted in an accelerated weathering tester (QUV/Spray, Q-Lab Corporation, USA) according to ASTM G 154 standard for 168 h to assess the durability of B-glass under harsh environment. Each cycle comprises an 8-hour UV exposure phase (340 nm, 60 ^o^C) and a 4-hour water spray phase (50 ^o^C), the total accelerated aging time was 168 hours, equivalent to 14 cycles.

RTP performance

Phosphorescence spectra and lifetime decay curves were recorded using a FLS1000 photoluminescence spectrometer (Edinburgh Instruments, Livingston, UK) equipped with a xenon lamp and a one-microsecond lamp (detector: photoelectric multiplier, 200nm < λ < 1700 nm). Afterglow emission spectra were recorded after a 10 ms delay.

2D WAXS analysis

Two dimensional wide angle X-ray scattering (2D-WAXS) measurements were performed using a Rigaku HomeLab diffractometer (Rigaku, Japan) with Cu Kα radiation (λ = 1.5405 Å). Fit-2D software was used to integrate azimuthal intensity distributions from the two-dimensional images. A Gaussian fitting of the profile was performed. All data were fitted with the correlation coefficients R^2^ above 0.99. Crystallite size of samples were calculated following to the Scherrer equation (1):

D (nm) = Kλ/βcosθ (1)

where K = 1.0 is the shape correction factor, λ = 0.154 nm is the radiation wavelength, β is the half width of diffraction peak, and θ is the diffraction angle.

The crystalline index (CrI) of different BCFs was calculated by the commonly employed peak-height method,^[1]^ according to the equation (2):

CrI = (I_200_-I_am_)/I_200_ (2)

where I_200_ is the intensity of the 200 peak at about 2θ = 22.0 ° after subtracting the background signal, I_am_ represents the intensity of the amorphous region at about 2θ = 18 °.

Simulation methods

All calculations were carried out with the Gaussian 16 software.^[2]^ The B3LYP functional was adopted for all calculations in combination with the D3BJ dispersion correction.^[3,4]^ For geometry optimization, the 6-31g(d,p) basis set was used.^[5]^ The inter-molecular interactions were decomposed using the sobEDA energy decomposition method at the same level based on the optimized energy. The total interaction energy was divided into electrostatic interaction, dispersion interaction, induction interaction, and exchange interaction. Visualization is completed using VESTA.^[6]^


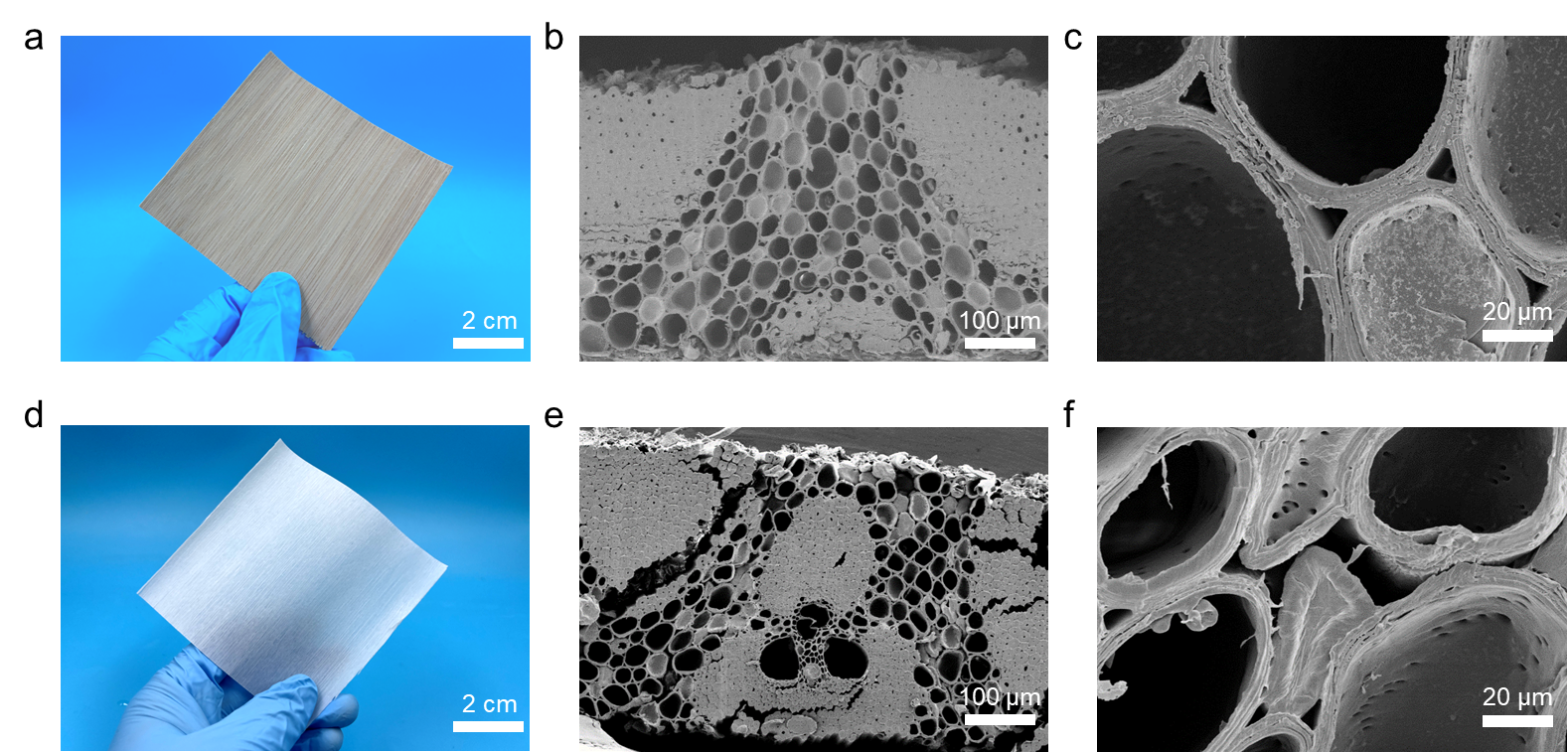


**Figure S1.** **Characterization of bamboo and delignified bamboo.** (a) Optical image of a bamboo veneer. (b, c) SEM image of cross section of bamboo. Fiber cells featured with thick cell wall assembled densely to form a macrofiber bundle (b) and parenchyma cells with thin cell wall and hollow structure (c) encompass the macrofiber bundle as a matrix. (d) Optical image of a delignified bamboo veneer. (e, f) SEM image of cross section of delignified bamboo. Many cracks and pores appeared in the macrofiber bundles (e) and the parenchyma cells were separated (f), which facilitates the impregnation of polymer.

**Figure S2.** Haze of B-glass.

**Figure S3.** Stress-strain curves of Bamboo and B-glass.


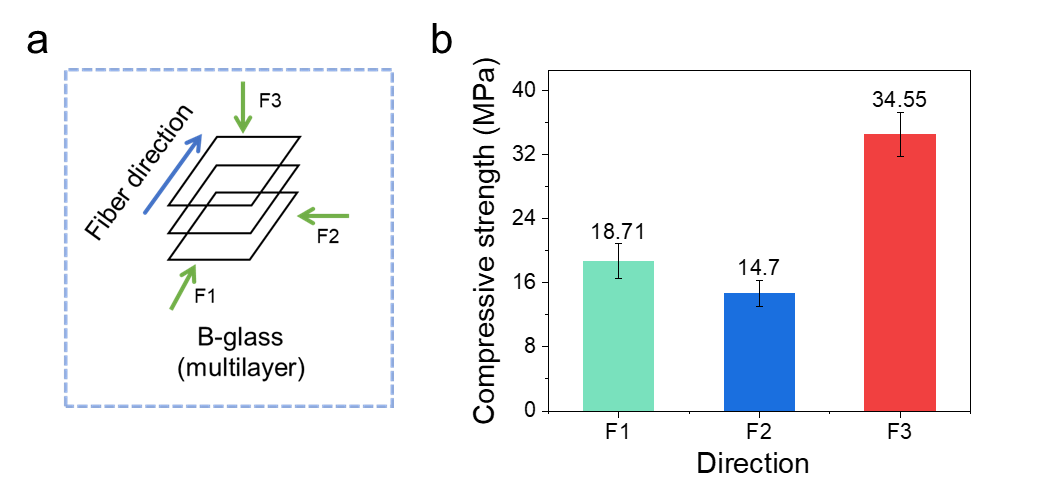


**Figure S4**. (a) Illustration of force direction and fiber directions. (b) Compressive strengths of B-glass.


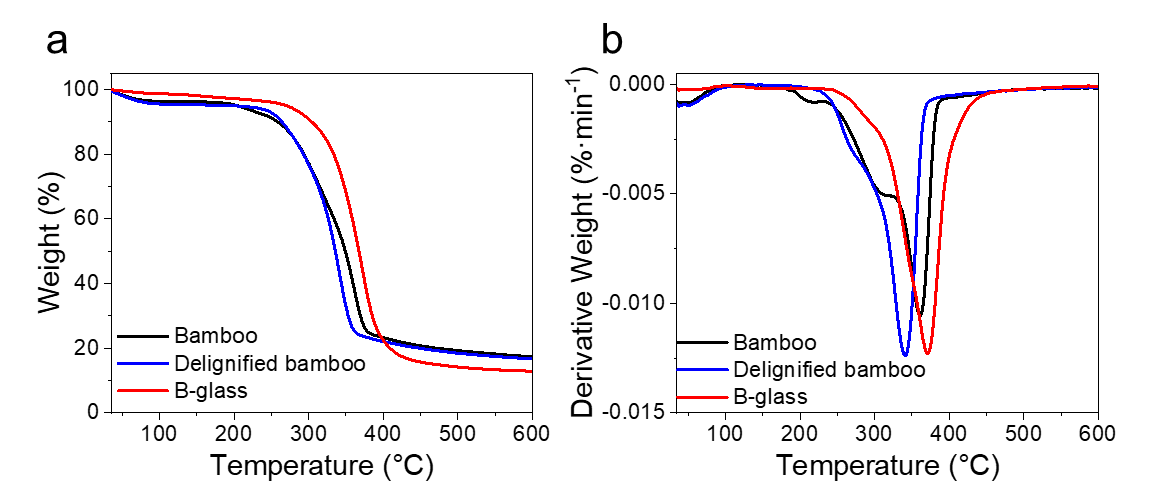


**Figure S5**. (a) TG and (b) DTG curves for natural bamboo, delignified bamboo and B-glass in a nitrogen atmosphere.


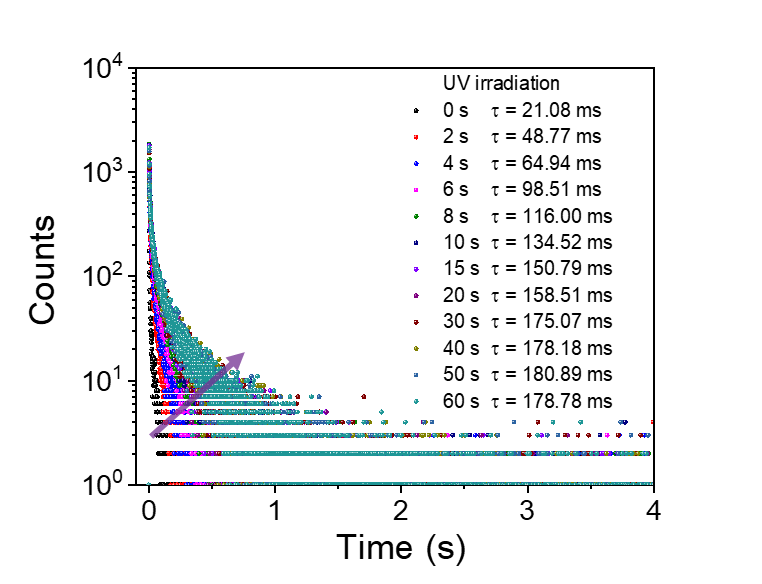


**Figure S6.** RTP lifetime of B-glass upon UV irradiation for different times.


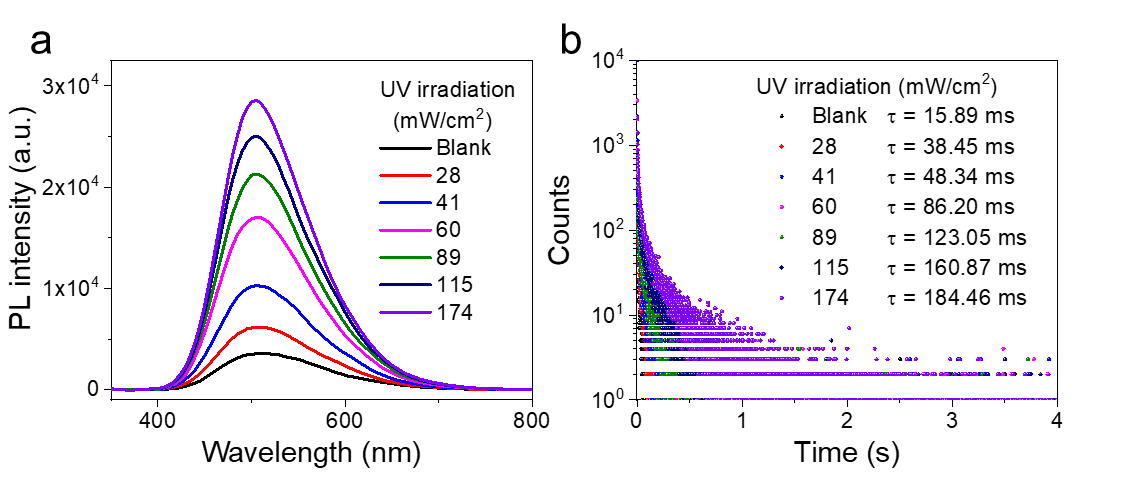


**Figure S7**. The photoactivated RTP performances of B-glass under different ultraviolet light intensities. (a) Phosphorescence spectra. (b) RTP decay profiles.

**Figure S8.** Fluorescence and RTP spectra of B-glass after UV irradiation.

**Figure S9.** Excitation-dependent RTP emission of B-glass after UV irradiation.


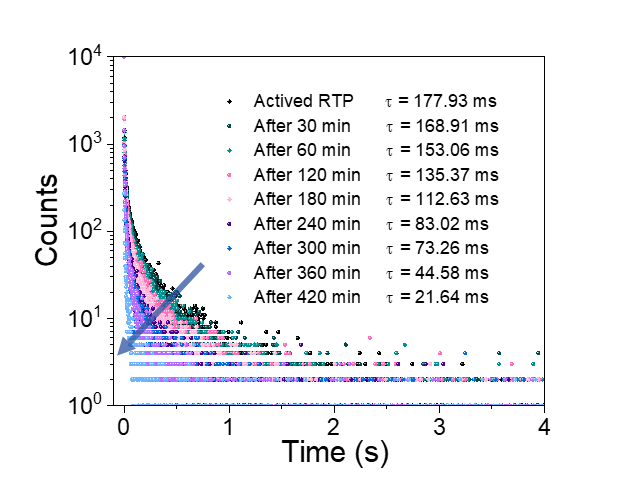


**Figure S10.** RTP lifetime of B-glass upon UV irradiation and RTP lifetime of B-glass activated by UV irradiation in an ambient environment for different times.


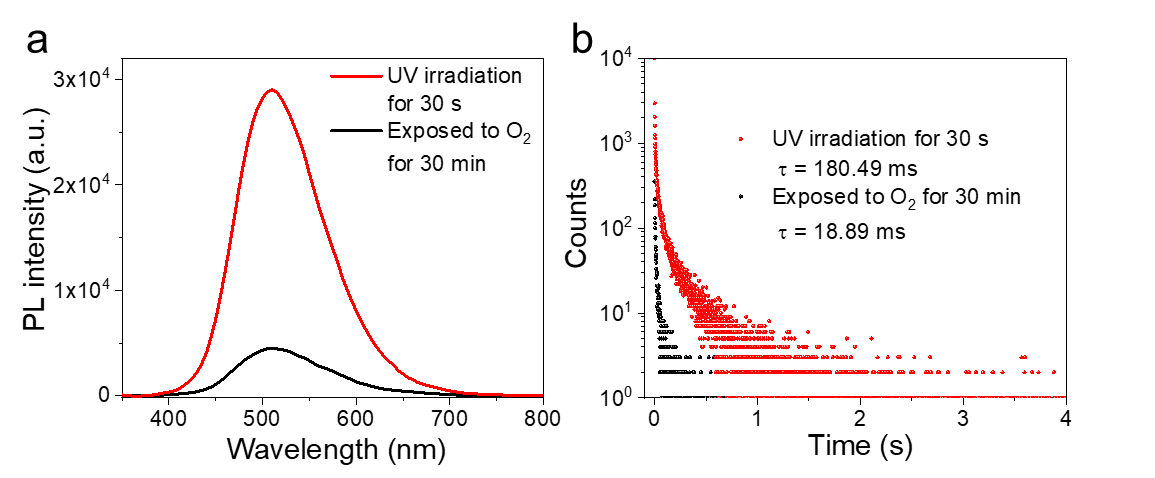


**Figure S11**. The RTP performance of photoactivated B-glass exposed to an oxygen-rich atmosphere. (a) Phosphorescence spectra. (b) RTP decay profiles.


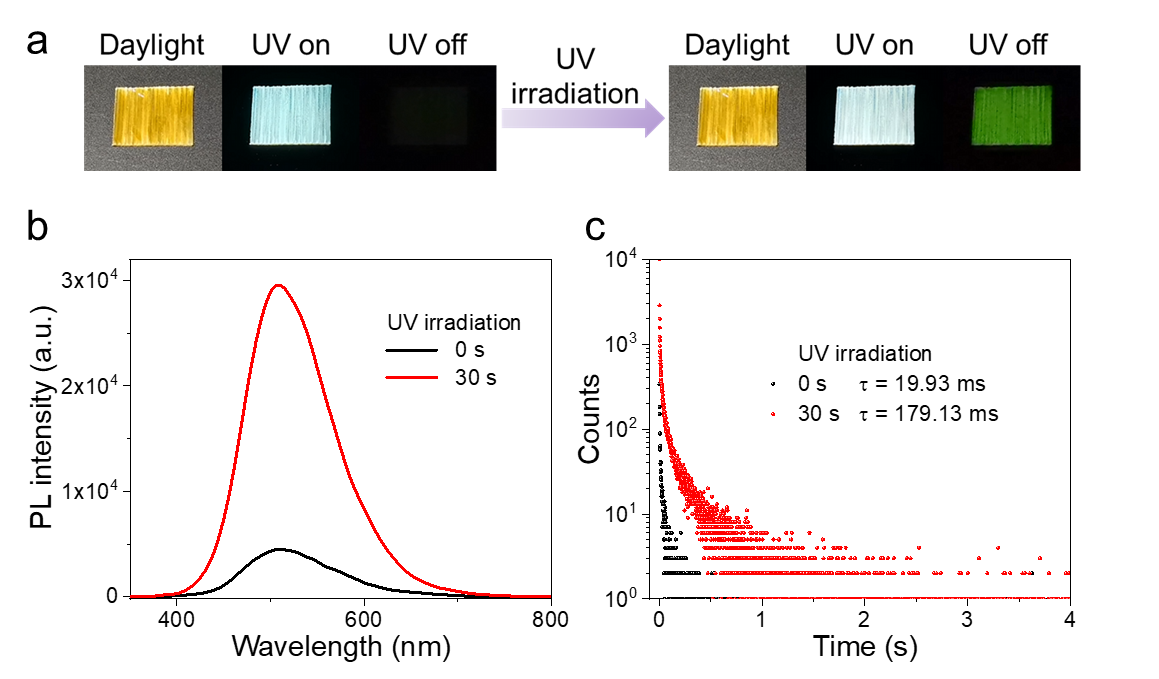


**Figure S12**. The photoactivated RTP performance of B-glass after 168 h weathering test. (a) Images of B-glass after 168 h weathering test upon UV irradiation and after removing the UV excitation. (b) Phosphorescence spectra. (c) RTP decay profiles.


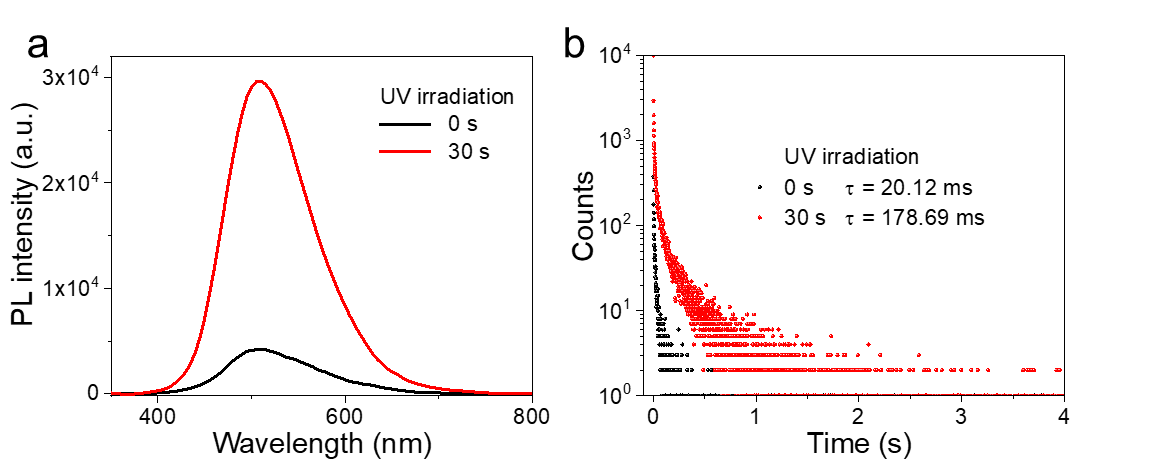


**Figure S13**. The photoactivated RTP performance of B-glass after 3-month air exposure. (a) Phosphorescence spectra. (b) RTP decay profiles.


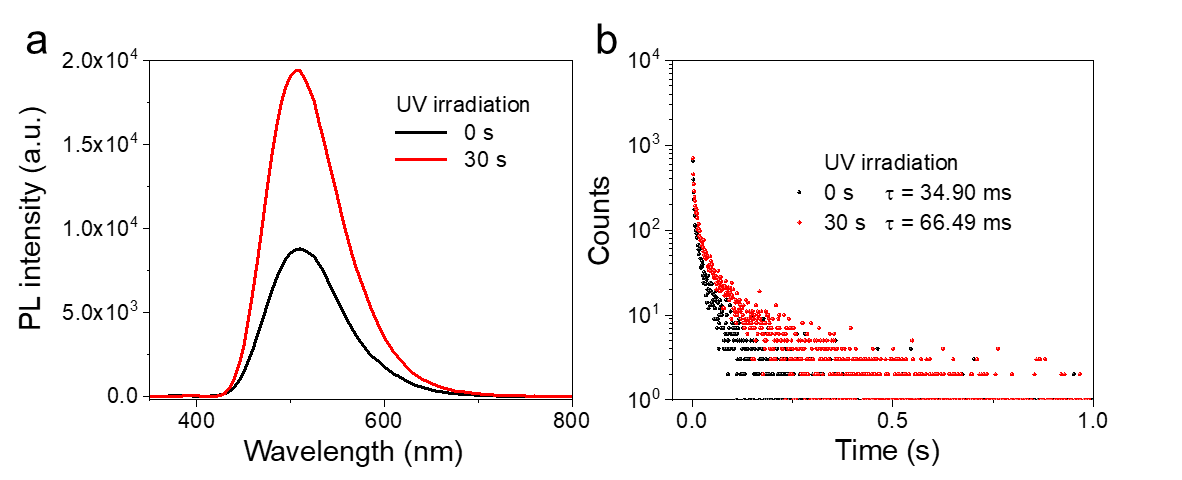


**Figure S14**. (a) Phosphorescence spectra of delignified bamboo/PMMA before and after UV irradiation. (b) RTP performance of delignified bamboo/PMMA before and after UV irradiation.

**Figure S15**. Comparison of RTP lifetime between B-glass (☆) and the reported materials. (TMA^[7]^, Eu^3+^@Zn-MOF^[8]^, PJJ-2^[9]^, MOF-5^[10]^, LIFM-SHL-2^[11]^, LIFM-130^[12]^, CDs@TEOS^[13]^, S-CDs^[14]^, CDs@Gelatin film^[15]^, C-Dots^[16]^, AA-CDs^[17]^, CNDs^[18]^, AN-CPDs^[19]^, CDs@MnAPO-CJ50^[20]^, RP-CDs^[21]^ and CDs@Zn-CHA^[22]^).


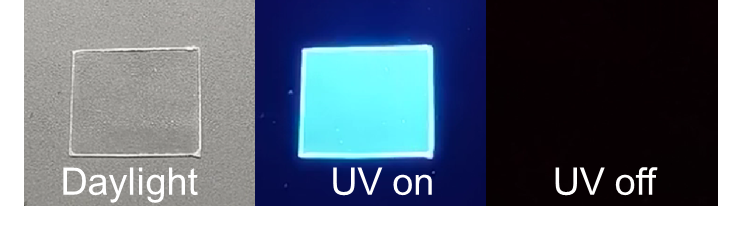


**Figure S16.** Photos of E-glass under daylight (left), UV light on (middle) and UV light off (right).

**
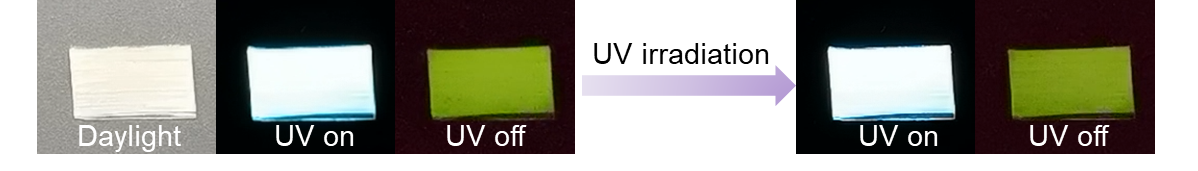
**

**Figure S17.** Images of delignified bamboo upon UV irradiation and after removing the UV excitation, Images of UV-activated delignified bamboo upon UV irradiation and after removing the UV excitation.


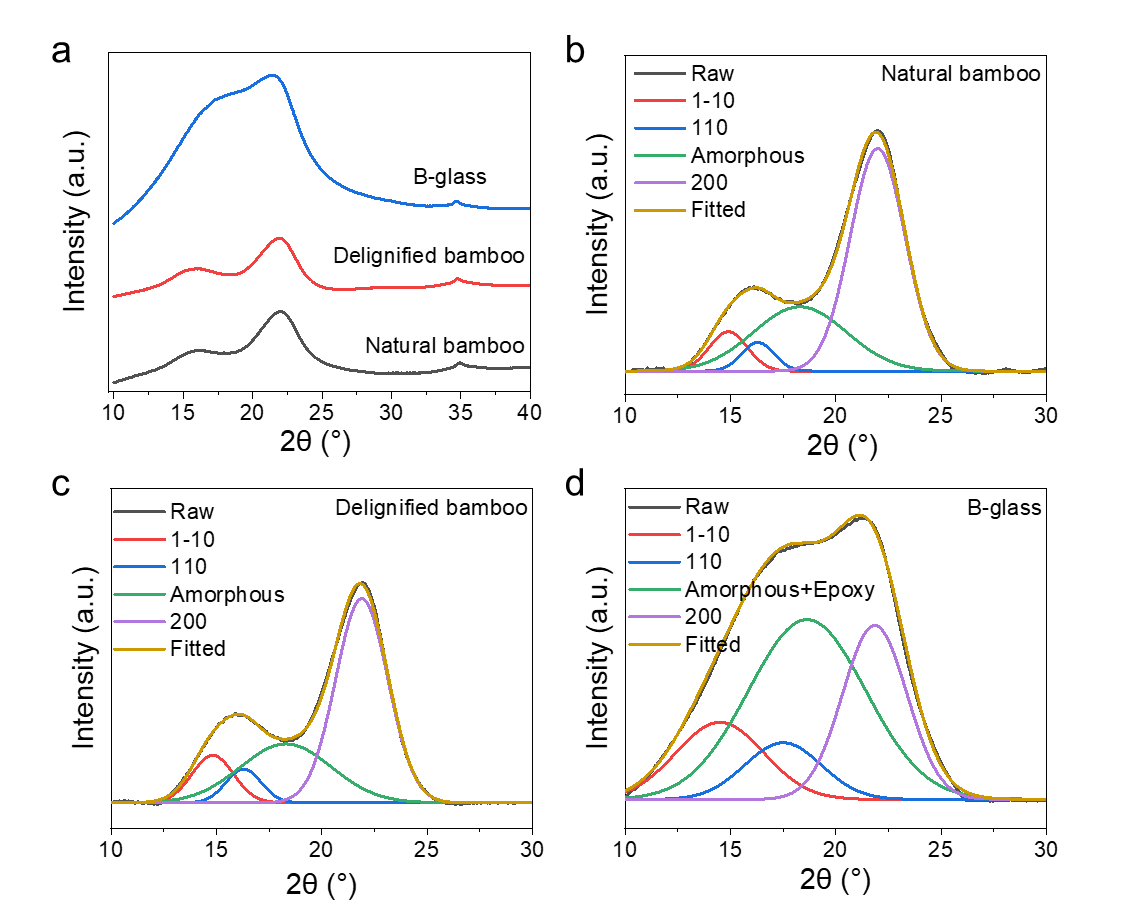


**Figure S18.** **Analysis of crystallite structure of bamboo, delignified bamboo, and B-glass**. (a) One-D integration of the azimuthal intensity of samples. (b-d) Gaussian fitting results in the range of 10°-30°from 1D integration profiles of bamboo (b), delignified bamboo (c), and B-glass (d).

**Figure S19**. The photo-activation time and RTP lifetime of B-glass with different thicknesses.


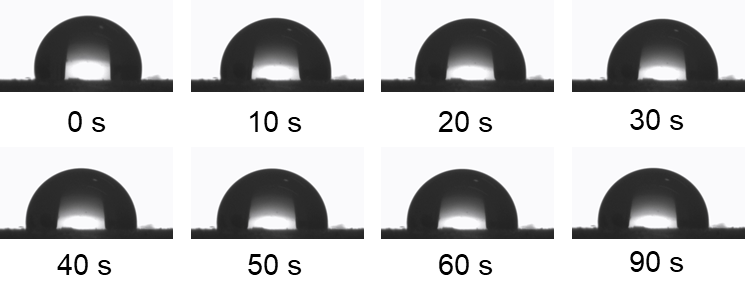


**Figure S20.** The contact angle of B-glass from 0 s to 90 s.


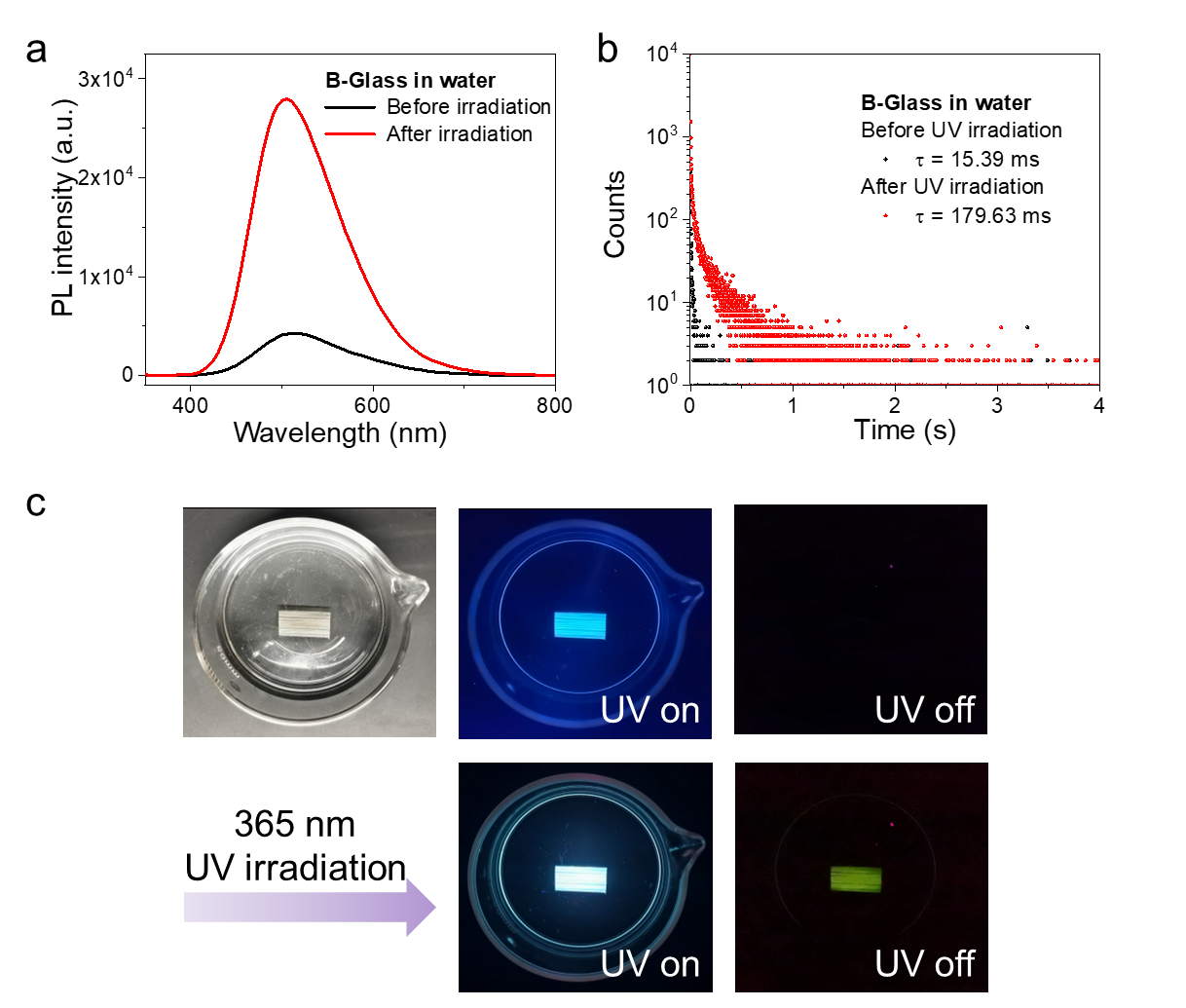


**Figure S21.** **Optical properties of B-glass in water.** (a) Phosphorescence spectra of B-glass before and after UV irradiation in water. (b) RTP performance of B-glass before and after UV irradiation in water. (c) Optical images of B-glass in water upon UV irradiation and after removing the UV excitation.

**Figure S22.** EPR spectra of B-glass in the presence of TEMP in the dark and upon UV irradiation at Ar atmosphere.

**Figure S23.** Phosphorescence spectra of B-glass irradiated in air and Ar.


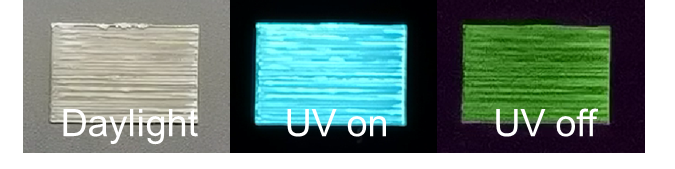


**Figure S24.** Photos of PVA-bamboo under daylight (left), UV light on (middle) and UV light off (right).


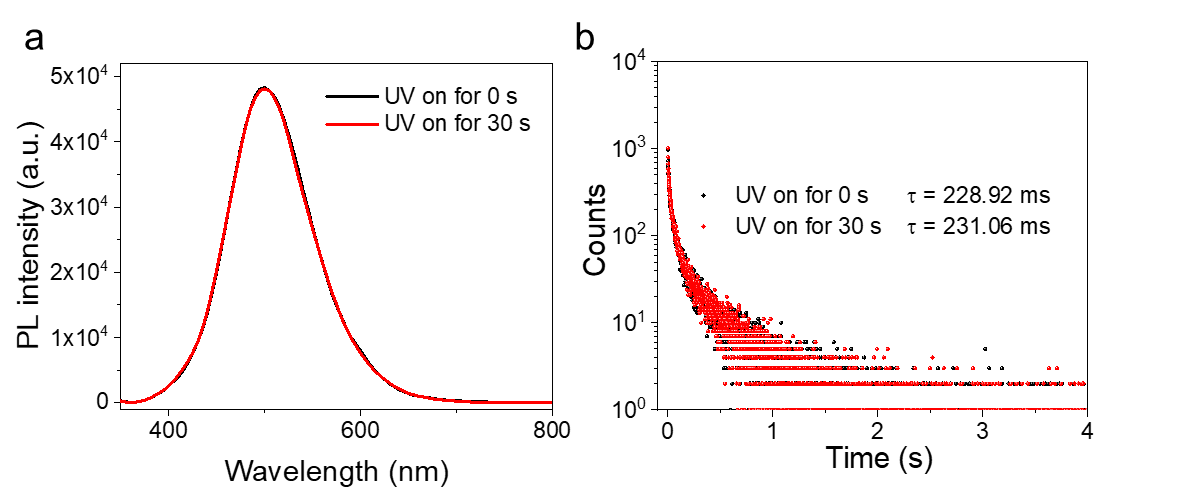


**Figure S25.** **RTP performances of PVA-bamboo composite.** (a) Phosphorescence spectra of PVA-bamboo composite before and irradiated in air for 30 s. (b) RTP lifetime PVA-bamboo composite before and irradiated in air for 30 s.


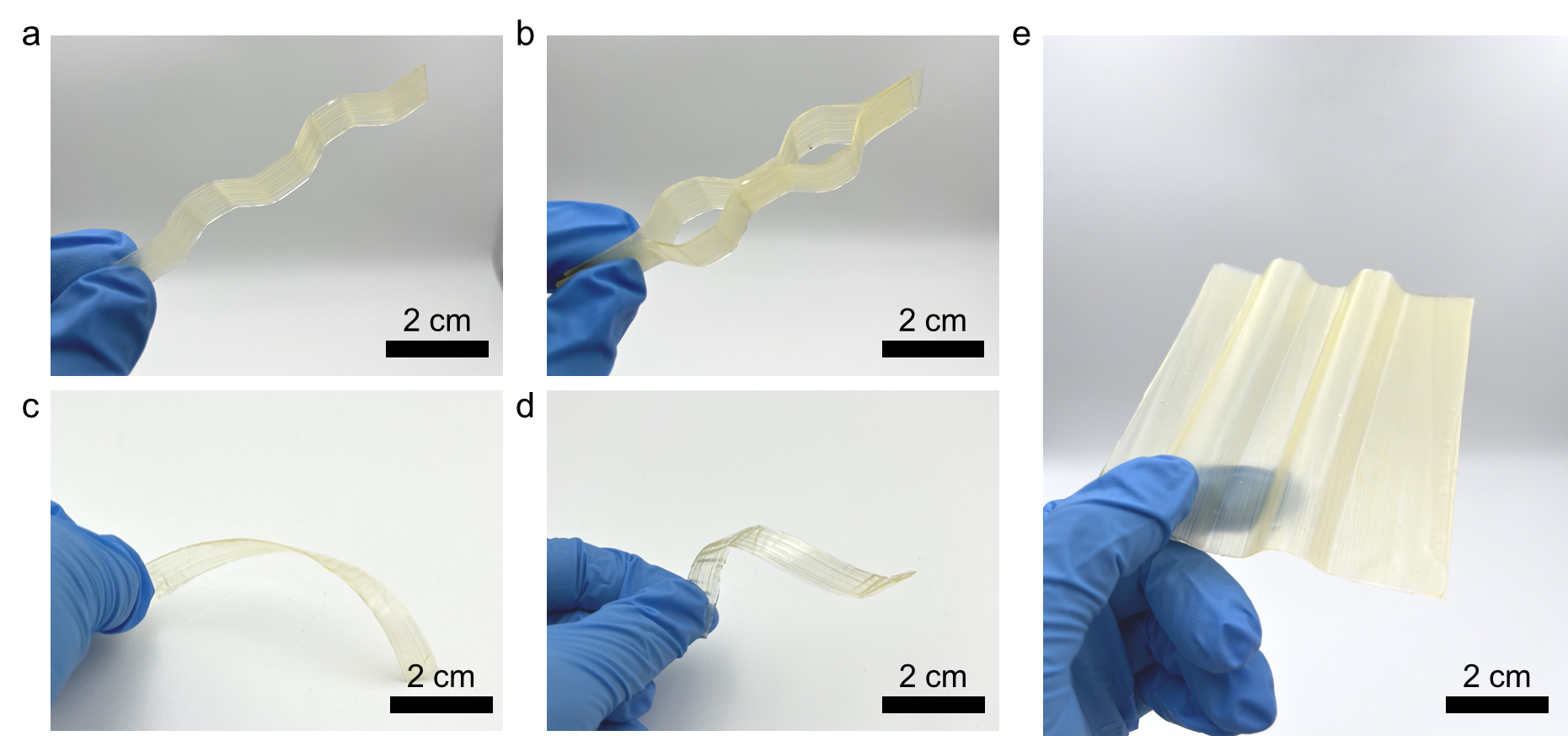


**Figure S26. Photograph of different shapes of B-glass.** (a) A piece of corrugated shaped B-glass. (b) Two pieces of corrugated shaped B-glass strips formed a hexagonal honeycomb structure. (c-d) Spiral shaped B-glass strips. (f) A corrugated shaped B-glass.


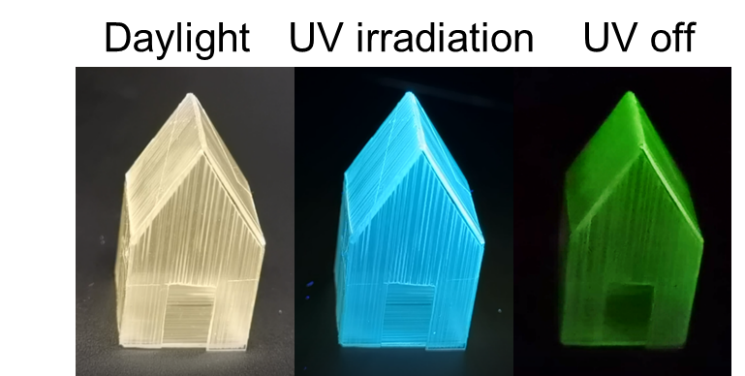


**Figure S27**. Photograph of a 3D house made of B-glass.

**Table S1.** Gaussian fitting results from the 1-D integration profile and the crystallite structure of natural bamboo, delignified bamboo, and B-glass.

|  | Peak | Peak position (°) | FWHM (°) | D hkl (nm) | d hkl (nm) |
| --- | --- | --- | --- | --- | --- |
| Natural bamboo | (1-10) | 14.90 | 2.13 | 3.761 | 0.594 |
|  | (110) | 16.30 | 1.82 | 4.409 | 0.543 |
|  | (200) | 21.98 | 3.06 | 2.644 | 0.404 |
| Delignified bamboo | (1-10) | 14.83 | 2.33 | 3.444 | 0.597 |
|  | (110) | 16.28 | 1.96 | 4.096 | 0.544 |
|  | (200) | 21.95 | 3.01 | 2.688 | 0.405 |
| B-glass | (1-10) | 14.51 | 4.91 | 1.631 | 0.610 |
|  | (110) | 17.50 | 4.29 | 1.874 | 0.506 |
|  | (200) | 21.70 | 3.60 | 2.247 | 0.409 |

**Table S2.** Pore structure and photoactivated RTP properties of B-glass with differernt vacuum time.

|  | BET surface area (m^2^/g) | Pore Volume (m^3^) | Oxygen transmission rates (cm³·mm^-^²·24 h^-1^·0.1 MPa^-1^) | Photoactivated time (s) | RTP lifetime (ms) |
| --- | --- | --- | --- | --- | --- |
| B-glass-5 min | 0.1929 | 6.1×10^-5^ | 30.54 | 60 | 179.77 |
| B-glass-10 min | 0.1535 | 4.9×10^-5^ | 24.91 | 50 | 178.99 |
| B-glass-30 min | 0.0823 | 2.3×10^-5^ | 18.37 | 30 | 180.90 |

**Video S1.** RTP optical activation of B-glass.

**References:**

[1] L. Segal, J. J. Creely, A. E. Martin, C. M. Conrad, *Text. Res. J.* **1959**, *29*, 786.

[2] M. J. Frisch, G. W. Trucks, H. B. Schlegel, G. E. Scuseria, M. A. Robb, J. R. Cheeseman, G. Scalmani, V. Barone, G. A. Petersson, H. Nakatsuji, X. Li, M. Caricato, A. V. Marenich, J. Bloino, B. G. Janesko, R. Gomperts, B. Mennucci, H. P. Hratchian, J. V. Ortiz, A. F. Izmaylov, J. L. Sonnenberg, D. Williams-Young, F. Ding, F. Lipparini, F. Egidi, J. Goings, B. Peng, A. Petrone, T. Henderson, D. Ranasinghe, V. G. Zakrzewski, J. Gao, N. Rega, G. Zheng, W. Liang, M. Hada, M. Ehara, K. Toyota, R. Fukuda, J. Hasegawa, M. Ishida, T. Nakajima, Y. Honda, O. Kitao, H. Nakai, T. Vreven, K. Throssell, J. A. Montgomery, Jr., J. E. Peralta, F. Ogliaro, M. J. Bearpark, J. J. Heyd, E. N. Brothers, K. N. Kudin, V. N. Staroverov, T. A. Keith, R. Kobayashi, J. Normand, K. Raghavachari, A. P. Rendell, J. C. Burant, S. S. Iyengar, J. Tomasi, M. Cossi, J. M. Millam, M. Klene, C. Adamo, R. Cammi, J. W. Ochterski, R. L. Martin, K. Morokuma, O. Farkas, J. B. Foresman, D. J. Fox, M. Gaussian, Inc., Wallingford CT, **2016**.

[3] A. D. Becke, *Phys. Rev. A* **1988**, *38*, 3098.

[4] S. Grimme, J. Antony, S. Ehrlich, H. Krieg, *J. Chem. Phys.* **2010**, *132*, 154104.

[5] T. Lu, Q. Chen, *J. Phys. Chem. A* **2023**, *127*, 7023.

[6] K. Momma, F. Izumi, *J. Appl. Crystallogr.* **2011**, *44*, 1272.

[7] H. Liu, W. Ye, Y. Mu, H. Ma, A. Lv, S. Han, H. Shi, J. Li, Z. An, G. Wang, W. Huang. *Adv. Mater.* **2022**, *34*, 2107612.

[8] D. Yang, H. Zheng, Y. Fang, Q. Liang, Q. Han, Y. Shi, X. Zheng. *Inorg. Chem.* **2022**, *61*, 7513.

[9] Z. Wang, J. Pan, X. Chen, M. Li, M. Pan. *Chem. Eng. J.* **2025**, *509*, 161076.

[10] X. Yang, D. Yan. *Chem. Sci.* **2016**, *7*, 4519.

[11] H. Sun, Z. Wang, R. Huang, Z. Chen, M. Pan, *Chem. Eng. J.* **2025**, *506*, 160075.

[12] J. Mo, Z. Wang, X. Xu, F. Dieter, M. Pan, C. Su, *Chem. Eng. J.* **2024**, *483*, 149343.

[13] B. Wang, Z. Sun, J. Yu, G. I. N. Waterhouse, S. Lu, B. Yang, *Smartmat* **2022**, *3*, 337.

[14] H. Sun, L. Zhou, R. Gong, M. Zhang, S. Shen, M. Liu, C. Wang, X. Xu, Z. Li, J. Cheng, W. Chen, L. Zhu, *ACS Appl. Mater. Interfaces* **2023**, *15*, 22415.

[15] R. Li, Y. Wang, Q. Li, G. Sun, *J. Lumin.* **2023**, *257*, 119725.

[16] W. Zou, T. Chen, D. Lin, W. Kong, W. Li, F. Xie, Q. Qu, Y. Wang, C. Jiang, *ACS Appl. Electron. Mater.* **2021**, *3*, 2661.

[17] S. Hu, K. Jiang, Y. Wang, S. Wang, Z. Li, H. Lin, *Nanomaterials* **2020**, *10* ,464.

[18] S. Song, K. Liu, Q. Cao, X. Mao, W. Zhao, Y. Wang, Y. Liang, J.-H. Zang, Q. Lou, L. Dong, C. Shan, *Light Sci. Appl.* **2022**, *11*, 146.

[19] C. Xia, S. Zhu, S.-T. Zhang, Q. Zeng, S. Tao, X. Tian, Y. Li, B. Yang, *ACS Appl. Mater. Interfaces* **2020**, *12*, 38593.

[20] B. Wang, Y. Yu, H. Zhang, Y. Xuan, G. Chen, W. Ma, J. Li, J. Yu, *Angew. Chem. Int. Ed.* **2019**, *58*, 18443.

[21] A. Kumar, R. Kumari, K. Negi, S. K. Sahu, *New J. Chem.* **2023**, *47*, 17110.

[22] B. Wang, Y. Mu, H. Zhang, H. Shi, G. Chen, Y. Yu, Z. Yang, J. Li, J. Yu, *ACS Cent. Sci.* **2019**, *5*, 349.
